# Varlitinib Downregulates HER/ERK Signaling and Induces Apoptosis in Triple Negative Breast Cancer Cells

**DOI:** 10.3390/cancers11010105

**Published:** 2019-01-17

**Authors:** Chun-Yu Liu, Pei-Yi Chu, Chun-Teng Huang, Ji-Lin Chen, Hsiu-Ping Yang, Wan-Lun Wang, Ka-Yi Lau, Chia-Han Lee, Tien-Yun Lan, Tzu-Ting Huang, Po-Han Lin, Ming-Shen Dai, Ling-Ming Tseng

**Affiliations:** 1 Comprehensive Breast Health Center, Taipei Veterans General Hospital, No.201, Sec.2, Shih-Pai Rd., Taipei 112, Taiwan; jlchen_@outlook.com (J.-L.C.); rabbitwang78tw@gmail.com (W.-L.W.); cayeelau@gmail.com (K.-Y.L.); gahanleeo@gmail.com (C.-H.L.); pj165210@gmail.com (T.-Y.L.); abby-eve@hotmail.com (T.-T.H.); lmtseng@vghtpe.gov.tw (L.-M.T.); 2 Division of Medical Oncology, Department of Oncology, Taipei Veterans General Hospital, No.201, Sec.2, Shih-Pai Rd., Taipei 112, Taiwan; huangchunteng@gmail.com (C.-T.H.); synthia730513@gmail.com (H.-P.Y.); 3 School of Medicine, National Yang-Ming University, No.155, Sec.2, Li-Nong Street, Taipei 112, Taiwan; 4 Division of Transfusion Medicine, Department of Medicine, Taipei Veterans General Hospital, No.201, Sec.2, Shih-Pai Rd., Taipei 112, Taiwan; 5 Department of Pathology, Show Chwan Memorial Hospital, No.542, Sec.1, Chung-Shan Rd., Changhua City 500, Taiwan; chu.peiyi@msa.hinet.net; 6 School of Medicine, Fu Jen Catholic University, No.510, Zhong-zheng Rd., Xin-zhuang Dist., New Taipei City 24205, Taiwan; 7 Division of Hematology and Oncology, Department of Medicine, Yang-Ming Branch of Taipei City Hospital, No.145, Zhengzhou Rd., Datong Dist., Taipei 103, Taiwan; 8 Department of Medical Genetics, National Taiwan University Hospital, No.7, Chung-Shan South Rd., Taipei 100, Taiwan; pohanlin01@gmail.com; 9 Graduate institute of medical genomics and proteomics, National Taiwan University, No.1, Sec.4, Roosevelt Rd., Taipei 106, Taiwan; 10 Hematology/Oncology, Tri-Service General Hospital, National Defense Medical Center, No.325, Sec.2, Cheng-gong Rd., Taipei 114, Taiwan; dms1201@gmail.com; 11 Department of Surgery, Taipei Veterans General Hospital, Taipei 112, Taiwan

**Keywords:** pan-HER inhibitor, triple-negative breast cancer

## Abstract

Triple-negative breast cancer (TNBC) is a complex disease associated with the aggressive phenotype and poor prognosis. TNBC harbors heterogeneous molecular subtypes with no approved specific targeted therapy. It has been reported that HER receptors are overexpressed in breast cancer including TNBC. In this study, we evaluated the efficacy of varlitinib, a reversible small molecule pan-HER inhibitor in TNBC. Our results showed that varlitinib reduced cell viability and induced cell apoptosis in most TNBC cell lines but not in MDA-MB-231 cells. MEK and ERK inhibition overcame resistance to varlitinib in MDA-MB-231 cells. Varlitinib inhibited HER signaling which led to inhibition of migration, invasion and mammosphere formation of TNBC cells as well as significant suppression of tumor growth of MDA-MB-468 xenograft mouse model. In summary, these results suggest that HER signaling plays an important role in TNBC progression and that pan-HER inhibition is potentially an effective treatment for TNBC patients.

## 1. Introduction

Triple-negative breast cancer (TNBC) is well-known for its aggressive phenotype and poor prognosis with genetic, transcriptomic and histological heterogeneity [1]. Due to the lack of estrogen receptor (ER), progesterone receptor (PR) and human epidermal growth factor receptor 2 (HER2) amplification, TNBC patients do not respond to hormonal- and trastuzumab-based targeted therapies. Current standard treatment for patients with early-stage TNBC includes cytotoxic chemotherapy with taxane- and anthracycline-based combination therapy, but the prognosis is poor [2]. Several clinical studies reported that platinum-based agents used as the first-line of treatment for patients with TNBC resulted in longer progression-free survival, but eventually also led to the development of drug resistance [3,4]. There has been no targeted therapy approved for TNBC. The heterogeneous molecular subtypes of TNBC present an opportunity for identification of novel therapeutic avenues.

The human HER receptor family has four receptor tyrosine kinases: EGFR (HER1), HER2, HER3 and HER4. HER receptor comprises of an extracellular ligand binding domain, a transmembrane domain, as well as an intracellular protein kinase domain and carboxyl terminal tail [5]. Activation of HER signaling results in mitogen activated protein kinase (MAPK) and phosphoinositide 3-kinase (PI3K)/Akt pathways upregulation, which regulate several cellular processes such as proliferation, apoptosis and metastasis [6]. It has been reported that expression levels of HER family in TNBC are diverse but HER2, HER3 and HER4 transcripts in TNBC are significantly lower than non-TNBC [7]. Nakai et al. reviewed that EGFR expression range from 13% to 76% in TNBC. EGFR overexpression was correlated with poor prognosis and drug resistance [8]. Approximately 41.2% of TNBC cohort was positive staining for HER3, which linked poorer disease free survival (DFS) and overall survival than HER3-negative TNBC subset [9]. HER4 expression has also been reported to be associated with poor prognosis in TNBC [7]. Pan-HER inhibition is considered to be a potential treatment for solid tumor patients including breast, biliary, colorectal, and non-small cell lung cancer [10,11,12]. Several pan-HER inhibitors such as afatinib, dacomitinib, and neratinib have shown promising results in TNBC in vitro [13,14,15]. All of these molecules are irreversible inhibitors of HER receptors and have been observed with a relatively high incidence of GI side effects such as diarrhea [16].

Varlitinib (formally known as ASLAN001) is a highly potent, reversible, oral nanomolar small molecule pan-HER inhibitor of the receptor tyrosine. Both in vitro and in vivo experiments have been conducted to demonstrate varlitinib’s efficacy [17]. In addition, two varlitinib clinical trials in patients with HER2 positive metastatic breast cancer have been conducted and showed signs of clinical activity [18] (ClinicalTrials.gov Identifier: NCT02338245 and NCT02396108). Given the possible link between TNBC and HER signaling, we sought to study the activity of varlitinib in TNBC cell lines, to determine whether this could be a novel therapeutic opportunity.

## 2. Results

### 2.1. Varlitinib Exerts Anti-Proliferation Ability and Induces Apoptosis

**Figure 1:**
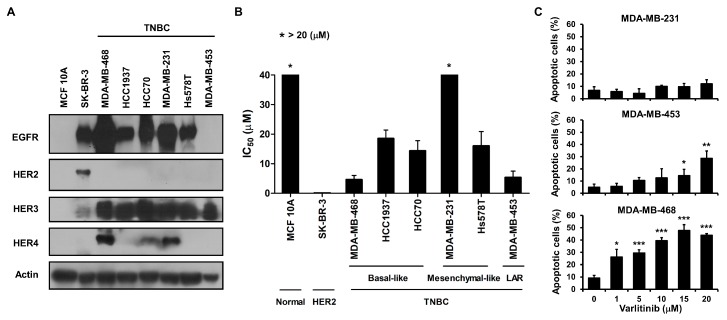
Varlitinib exerts anti-proliferation ability and induces apoptosis in MDA-MB-453 and MDA-MB-468 cells but not MDA-MB-231 cells. (**A**) Whole-cell extracts were analyzed by western blot analysis using antibodies against anti-EGFR, anti-HER2, anti-HER3, anti-HER4 and anti-β-actin. (**B**) Cells were treated with various concentrations of varlitinib for 72 h for MTT assay. IC_50_ values were determined and shown. (**C**) MDA-MB-231, MDA-MB-453 and MDA-MB-468 cells were treated with various concentrations of varlitinib for 72 h. The treated cells were analyzed using flow cytometry. The means ± SEM of three independent experiments performed in triplicate are shown. Student’s *t*-test, * *p* < 0.05, ** *p* < 0.01, *** *p* < 0.001.

We first examined the endogenous expression levels of HER receptors in TNBC cell lines, HER2-amplified SK-BR-3 cells as well as MCF 10A breast epithelial cell line. Results of western blot analysis showed that most of TNBC cell lines expressed higher levels of EGFR and HER3 than MCF 10A cells. TNBC cell lines were deficient in HER2 and differentially expressed HER4 (Figure 1A). To explore varlitinib’s anti-tumor activity, cells were treated with varlitinib at various concentrations. Compared with MCF 10A cell lines, most of the cell lines exhibited lower IC_50_ except MDA-MB-231 cells (Figure 1B and Figure S1). Moreover, varlitinib significantly induced cell apoptosis in MDA-MB-453 and MDA-MB-468 cells but not in MDA-MB-231 cells (Figure 1B,C).

### 2.2. Varlitinib Suppresses MEK/ERK Pathway in TNBC Cells

**Figure 2:**
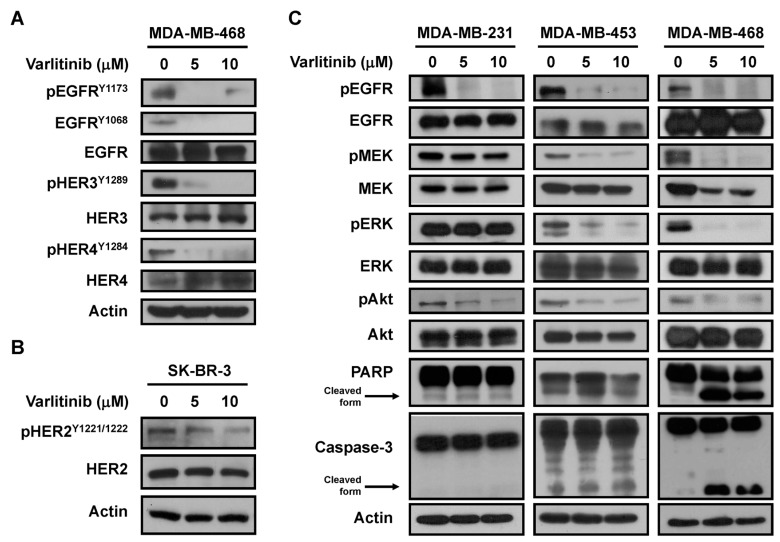
Varlitinib inhibits MEK/ERK and Akt pathway in TNBC cells. (**A**,**B**) Whole-cell extracts of SK-BR-3 and MDA-MB-468 cells treated with indicated concentration of varlitinib for 48 h were prepared for western blot analysis using antibodies against anti-phospho-EGFR^Y1173^, anti-phospho-EGFR^Y1068^, anti-EGFR, anti-phospho-HER2^Y1221/1222^, anti-HER2, anti-phospho-HER3^Y1289^, anti-HER3, anti-phospho-HER4^Y1284^, anti-HER4 and anti-β-actin. (**C**) The whole-cell extracts from MDA-MB-231, MDA-MB-453 and MDA-MB-468 cells treated with indicated concentration of varlitinib for 48 h were prepared for western blot analysis using antibodies against anti-phospho-EGFR, anti-EGFR, anti-phospho-MEK, anti-MEK, anti-phospho-ERK, anti-ERK, anti-phospho-Akt, anti-Akt, anti-PARP, anti-Caspase-3 and anti-β-actin.

To explore the clinical significance of HER family, we examined data from The Cancer Genome Atlas (TCGA) database. EGFR, HER2, HER3 and HER4 gene alterations, including copy number variation, mutation and mRNA dysregulation, in patients with breast cancer and TNBC, were analyzed. Results showed that most of TNBC patients harbored EGFR upregulation compared to HER2, HER3 and HER4 (Figure S2A). In addition, TNBC tumor tissues harbored higher pEGFR^Y1173^ and pHER3^Y1289^ than normal tissues (Figure S2B). To evaluate the pan-HER inhibitor capacity in vitro, we examined the phosphorylation of HER family in varlitinib-treated SK-BR-3 (a HER2-expressing breast cancer cell line) and MDA-MB-468 cells. Data showed varlitinib reduced pEGFR, pHER3 and pHER4 in MDA-MB-468 cells as well as reduced pHER2 in SK-BR-3 cells (Figure 2A,B). Activation of HER receptors leads to the activation of downstream pathways including RAS/RAF/MEK/ERK and PI3K/Akt signaling. Our western blot results demonstrated that varlitinib treatment inhibited EGFR, AKT, MEK and ERK activation in MDA-MB-453 and MDA-MB-468 cells. In addition, varlitinib treatment also resulted in increased levels of cleaved PARP and cleaved Caspase-3 in these TNBC cell lines. Conversely, varlitinib did not inhibit MEK/ERK signaling in MDA-MB-231 cells (Figure 2C). 

### 2.3. Varlitinib Induces Apoptosis through ERK Inhibition in TNBC Cells

**Figure 3:**
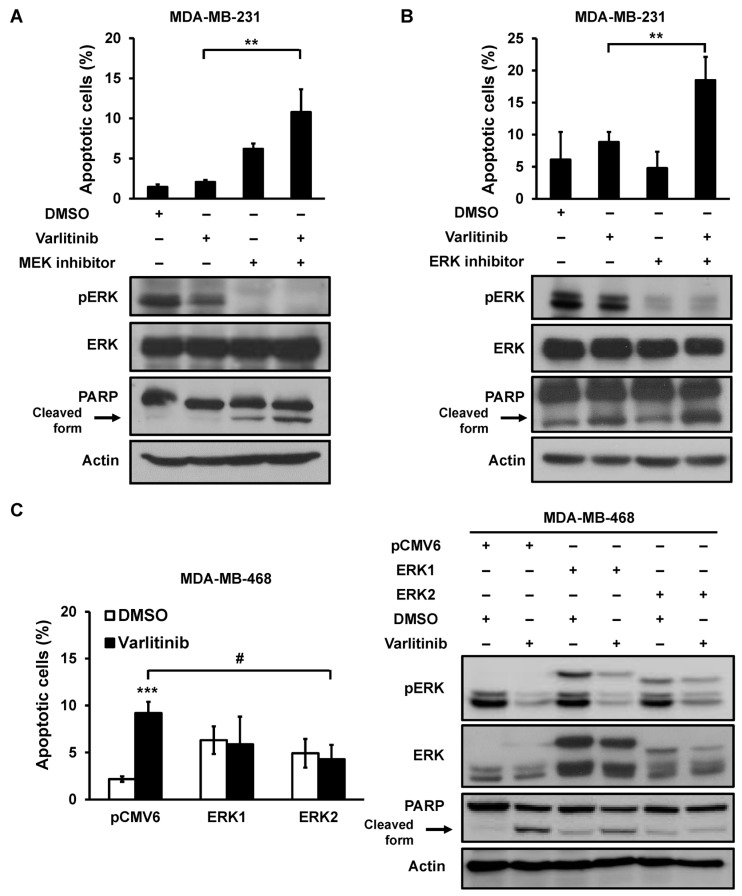
ERK signaling mediates varlitinib-induced apoptosis in TNBC cells. (**A**) MDA-MB-231 cells were treated with MEK inhibitor GDC-0973, varlitinib or DMSO for 48 h. (**B**) MDA-MB-231 cells were treated with ERK inhibitor SCH772984, varlitinib or DMSO for 48 h. (**C**) MDA-MB-468 cells were transfected with ERK1-, ERK2-expressing or control plasmids (pCMV6) for 24 h, and the transfected cells were further treated with varlitinib or DMSO for 48 h. Apoptosis in the treated cells was analyzed by flow cytometry (left) and the whole-cell extracts of treated cells were analyzed by western blot analysis using antibodies against anti-phospho-ERK, anti-ERK, anti-PARP and anti-β-actin (right). The means ± SEM of three independent experiments performed in triplicate are shown. Student’s *t*-test, ** *p* < 0.01, *** *p* < 0.001 compared with cells treated with DMSO control. # *p* < 0.05 compared with cells transfected with pCMV6 vector and treated with varlitinib.

RAF/MEK/ERK pathway has different effects on prevention of apoptosis, cell cycle arrest and induction of drug resistance [19]. Our results suggested that MEK/ERK activation play a role in resistance to varlitinib-induced apoptosis in MDA-MB-231 cells. MDA-MB-231 cells were treated with varlitinib in combination with either MEK inhibitor or ERK inhibitor. Results showed that varlitinib combination treatment with MEK/ERK inhibitors induced apoptosis and also increased the level of cleaved PARP in MDA-MB-231 cells (Figure 3A,B). In contrast, varlitinib-induced cell apoptosis or PARP cleavage were rescued by ERK2 overexpression but not ERK1 overexpression in MDA-MB-468 cells (Figure 3C). 

### 2.4. Varlitinib Inhibits Migration, Invasion and Mammosphere Formation of TNBC Cells

**Figure 4:**
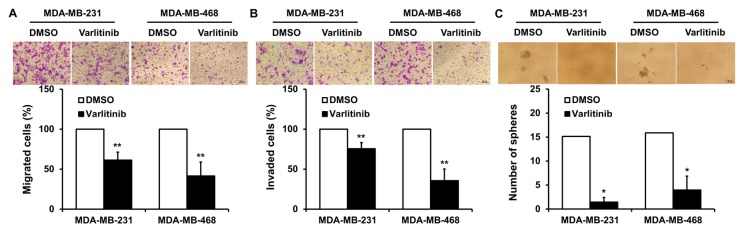
Varlitinib reduces the abilities of migration, invasion and mammosphere formation of TNBC cells. (**A**–**C**) MDA-MB-231 and MDA-MB-468 cells were treated with varlitinib or DMSO for subsequent migration (**A**), invasion (**B**) and mammosphere assays (**C**). The means ± SEM of three independent experiments performed in triplicate are shown (100× magnification times for A,B,C). Student’s *t*-test, * *p* < 0.05, ** *p* < 0.01.

To evaluate the role of varlitinib in cancer progression of TNBC cells, functional assays were performed. Our results showed that varlitinib treatment inhibited cell migration, invasion and mammosphere formation of MDA-MB-231 and MDA-MB-468 cells (Figure 4A–C). 

### 2.5. Varlitinib Shows Anti-Tumor Effect in TNBC Xenograft Model

**Figure 5:**
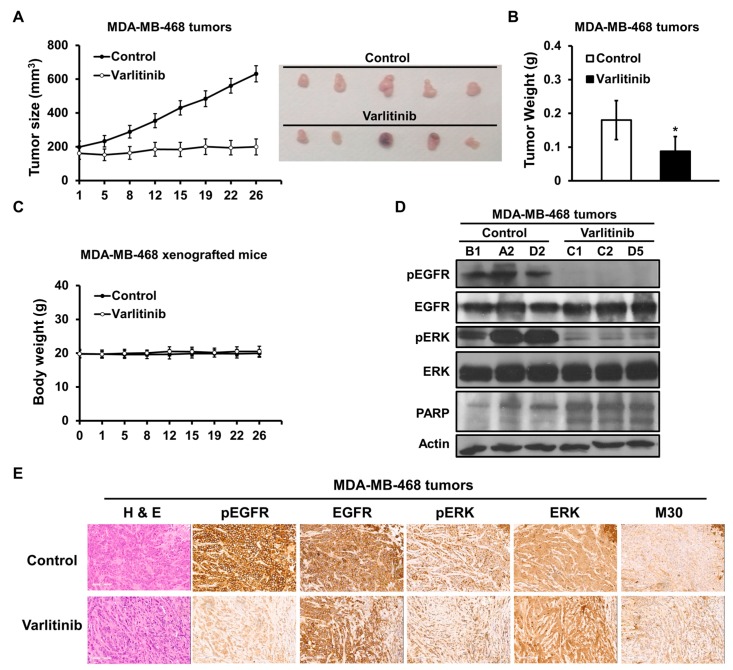
Varlitinib inhibits tumor growth of TNBC. (**A–E**) MDA-MB-468-bearing mice were treated with varlitinib at 100 mg/kg BID or vehicle and the (**A**) xenografted tumors sizes, (**B**) weights and (**C**) the body weights of xenografted mice were measured. (**D**) The xenografted tumors were analyzed by western blotting using antibodies against anti-pEGFR, anti-EGFR, anti-pERK, anti-ERK, anti-PARP and anti-β-actin. (**E**) Immunohistochemical staining of pEGFR, EGFR, pERK, ERK and M30 in MDA-MB-468 xenografts (200× magnification times for E). Student’s *t*-test, * *p* < 0.05. Data are shown as mean ± SD.

Nude mice were subcutaneously implanted with MDA-MB-468 cells to evaluate anti-tumor activity of varlitinib. Once xenograft tumor sizes reached 200 mm^3^ varlitinib were orally administered. Varlitinib suppressed tumor growth in MDA-MB-468 xenograft mice with no effect on body weight (Figure 5A–C). In comparison to the control group, varlitinib treatment significantly suppressed EGFR and ERK activation with increased PARP cleavage (Figure 5D). Immunohistochemical staining was performed to further examine protein expression and localization within the xenograft tumors. The results showed that varlitinib treatment reduced EGFR and ERK phosphorylation and elicited cell apoptosis with M30 staining (Figure 5D,E). These results demonstrated varlitinib exerting anti-tumor activity in TNBC via the inhibition of HER receptor and downstream signaling.

## 3. Discussion

HER receptors play a crucial role in breast cancer tumorigenesis including TNBC. EGFR, HER3 and HER4 expression in TNBC patients was associated with poor DFS [7,9,20]. These receptors might serve as prognostic markers and targets for treatments. However, several clinical studies demonstrated that EGFR inhibitors monotherapy and chemotherapy combination showed a partial response in TNBC [21,22]. The HER receptors form homodimers and heterodimers. Hetero-dimerization of EGFR with HER2, HER3 or HER4 might limit anti-tumor effect of EGFR inhibition, and pan-HER inhibitor could improve the efficacy of EGFR inhibition through limiting receptor cross-talk signaling [23]. 

Pan-HER inhibitors include afatinib, dacomitinib, and neratinib which induce irreversible inhibition [13,24]. U.S. Food and Drug Administration (FDA) approved afatinib for patients with metastatic non-small cell lung cancer whose tumors express EGFR exon 19 deletions or exon 21 (L858R) substitution mutations as well as the patients with advanced squamous cell lung cancer who have progressed after treatment with platinum-based chemotherapy [15]. In addition, FDA approved neratinib as extended adjuvant treatment for patients with early stage HER2 positive breast cancer. It has been reported that afatinib and neratinib inhibited cell growth of TNBC [25,26], however, TNBC patients have not shown significant improvement in clinical trials with these inhibitors [14,27]. 

The anti-tumor effects of varlitinib in TNBC were investigated in vitro and in vivo. From our experimental results, varlitinib suppressed cell viability in most of TNBC cells, however, MDA-MB-231 cells were resistant to apoptotic effect of varlitinib (Figure 1). Varlitinib is a reversible pan-HER inhibitor with nanomolar potency against HER1, HER2 and HER4 in cell-based assays of gastric cancer [17]. HER3 has defective tyrosine kinase activity. Upon hetero-dimerization, HER3 can be phosphorylated by EGFR or HER2. Therefore, a pan-HER inhibitor could also inhibit HER3 receptor indirectly [13]. Our data also indicated that varlitinib exhibited pan-HER inhibitor activity in breast cancer cells. HER signaling triggers RAS/RAF/MEK/ERK and PI3K/Akt signal pathways. We examined the downstream signaling and found that varlitinib inhibited activation of MEK and ERK in MDA-MB-453 and MDA-MB-468 but not in MDA-MB-231 cells (Figure 2).

In MDA-MB-468 cells, it was also shown that reactivation of ERK in combine with varlitinib repressed the cell apoptosis effect of varlitinib. It was shown that the combination of MEK or ERK inhibitors with varlitinib induced cell apoptosis in MDA-MB-231 cells (Figure 3). MDA-MB-231 cells harbored BRAF^G464V^ and KRAS^G13D^ mutations [28]. One study showed that exogenous expression of BRAF mutants including BRAF^G464V^ in SK-BR-3 cells treated with lapatinib, a HER1/HER2 tyrosine kinase inhibitor, did not reduce pERK level [29]. This may possibly be due to the drug resistance associated with KRAS mutations. Another study showed KRAS^G13D^ and KRAS^G12R^ mutations in drug resistance to cetuximab in vitro and in vivo [30,31]. These studies indicated that KRAS and BRAF mutations drove ERK dependent growth in various cancers. RAS/RAF/MEK/ERK pathway is activated by upstream molecules alteration. Mutations occurring in this pathway might lead to chemoresistance. Targeting this cascade is complicated and the presence of upstream mutations may result in pathway activation [32,33]. However, BRAF and KRAS mutations are rare in breast cancer [34] with BRAF and KRAS mutated in 0.45% and 0.54% of The Cancer Genome Atlas (TCGA) breast cancer genomes respectively.

Functional assays showed that varlitinib led to inhibition of migration, invasion and mammosphere formation in TNBC cells (Figure 4) regardless of their MEK and ERK status. Varlitinib inactivated Akt in TNBC cells shown in Figure 2. Several studies revealed that Akt pathway participated in cancer metastasis including breast cancer [35]. Akt activation up-regulates Snail, a transcriptional repressor of E-cadherin, leading to epithelial–mesenchymal transition (EMT) [36]. EMT elicits acquisition of motility and invasion and contributes to stemness [37,38]. A phase II LOTUS trial demonstrated that improved progression free survival in patients with metastatic TNBC receiving Akt inhibitor ipatasertib plus paclitaxel [39]. Our result shows varlitinib’s anti-tumor activities in both in vitro and in vivo experiments (Figure 4 and Figure 5). Varlitinib may exhibit anti-tumor activities not only through MEK/ERK inhibition but Akt dephosphorylation. However, the role of pan-HER inhibition in tumor metastasis in vivo should be further investigated.

To this date, several irreversible small molecule pan-HER inhibitors are in clinical trials, with diarrhea and skin toxicity as common adverse effects [40]. In general, reversible drugs are considered to be safer than irreversible drugs, and clinical experience with varlitinib shows a low degree of GI side effects. Clinical trial data with varlitinib in MBC patients failing trastuzumab showed promising activity compared to lapatinib [18].

## 4. Materials and Methods

### 4.1. Cell Culture, Reagents and Transfection

Human TNBC cell lines MDA-MB-231, MDA-MB-468, MDA-MB-453, HCC1937 and Hs578T and HER2-positive SK-BR-3 human breast cancer cells were cultured in Dulbecco’s Modified Eagle Medium (DMEM) with 10% fetal bovine serum. Human HCC70 TNBC cell lines and MCF 10A breast epithelial cell line were cultured in RPMI-1640 Medium with 10% fetal bovine serum. Cell lines were purchased from American Type Culture Collection (Manassas, VA, USA). For transfection, cells were seeded onto 6-well for 24 h and transiently transfected by Lipofectamine^TM^ 3000 Reagent (Thermo Fisher Scientific, Waltham, MA, USA). The MEK inhibitor GDC-0973 (Cobimetinib, Adooq BioScience, Irvine, CA, USA), ERK1/2 inhibitor SCH772984 (Selleck Chemicals, Houston, TX, USA) were used at the indicated concentrations in dimethyl sulfoxide (DMSO). Varlitinib for in vitro and in vivo experiments were generously provided by ASLAN Pharmaceuticals.

### 4.2. Western Blot Analysis

Whole cell extract was prepared using RIPA buffer (Thermo Scientific) with a Halt^TM^ Protease and Phosphatase Inhibitor Cocktail (Thermo Scientific) [15]. Laemmli’s sample buffer was added to the cell lysates and boiled at 95 °C for 5 min. The cell lysates were then analyzed by sodium dodecyl sulfate-polyacrylamide gel electrophoresis using antibodies against anti-EGF Receptor, anti-phospho-EGF Receptor (Tyr1068), anti-HER2/ErbB2, anti-HER3/ErbB3, anti-HER4/ErbB4, anti-phospho-MEK1/2 (Ser217/221), anti-MEK1/2, anti-phospho-p44/42 MAPK (Erk1/2) (Thr202/Tyr204), anti-p44/42 MAPK (Erk1/2), anti-Akt, anti-phospho-Akt (Ser473), anti-PARP, anti-Caspase-3 and anti-β-actin (Cell Signaling).

### 4.3. MTT Assay

Cells were seeded onto 96-well plates for 24 h and treated with indicated concentrations of varlitinib for 72 h. The treated cells were incubated with 0.5 mg/mL MTT (Sigma-Aldrich, St. Louis, MO, USA) in each well at 37 °C for 3 h. The violet MTT formazan precipitates were subsequently dissolved in 100 μL DMSO. The absorbance at 570 nm was measured on an UQuant reader.

### 4.4. Flow Cytometry Analysis

For apoptosis detection, 5 × 10^5^ cells were harvested and suspended in 200 μL 1× Binding Buffer containing 5 μL APC Annexin V (BD Biosciences, San Jose, CA, USA) and 4 μL of 0.2 mg/mL Propidium Iodide (PI). Cells were incubated for 15 min at room temperature protected from light. The apoptotic cells were measured by APC Annexin V and PI double staining and analyzed by flow cytometry.

### 4.5. Migration and Invasion Assays

The migration and invasion assays were performed in 24-well plate for 16 and 20 h respectively. 3 × 10^4^ cells in 200 μL of serum free medium were seeded onto upper Cell Culture Insert with 8 μm pores (Greiner Bio One, Kremsmünster, Austria) for migration assay and Matrigel matrix (Corning, New York, NY, USA) coated Cell Culture Insert for invasion assay respectively. The lower chamber contained 900 μL of complete medium. The migrated and invaded cells were fixed with methanol for 10 min and stained with 0.005% crystal violet for 1 h at room temperature. The numbers of migrated and invaded cells were counted under the microscope from 10 random fields.

### 4.6. Mammosphere Assay

The mammosphere formation assay was performed in 96-well Ultra-Low attachment plates (Corning). 500 cells were incubated in DMEM/F-12 medium containing 1% N-2, 2% B-27, 20 ng/mL bFGF and 20 ng/mL EGF (Gibco, Thermo Fisher Scientific) at 37 °C for 9 days and fresh medium was supplemented every 3 days. The mammospheres which exceed 50 μm in diameter were counted under the microscope.

### 4.7. Xenograft Tumor Growth

The animal experiments were approved by the Institutional Animal Care and Use Committee of Taipei Veterans General Hospital (IACUC No. 2017-073). Five-week-old female NCr athymic nude mice were obtained from the National Applied Research Laboratories National Laboratory Animal Center (Taipei, Taiwan). Mice were inoculated subcutaneously in the right flank with MDA-MB-468 cells (1.5 × 10^6^) suspended in 0.1 mL serum-free medium containing 30% matrigel (BD Biosciences) under isoflurane anesthesia. MDA-MB-468 tumor-bearing mice with mean tumor volume~200 mm^3^ were randomly divided into 2 groups (*n* = 10 per group) and treated with varlitinib at 100 mg/kg BID PO or vehicle (solutol HS15:propylene glycol:PBS = 25:25:50). Tumor sizes were measured using caliper and volumes calculated using a standard formula: width^2^ × length × 0.52. Tumors were harvested from mice at the time of sacrifice for further data analysis. All animal studies were performed in accordance with protocols approved by the Institutional Laboratory Animal Care and Use Committee of the Taipei Veterans General Hospital.

### 4.8. Immunohistochemical Staining

Primary antibodies against anti-pEGFR (ab40815, Abcam, Cambridge, MA, USA), anti-EGFR (Z2037, Zeta Corporation, Sierra Madre, CA, USA), anti-pERK (ab50011, Abcam), anti-ERK (ab17942, Abcam), and anti-M30 CytoDEATH (No. 10700, PEVIVA, West Chester, OH, USA) antibodies were used as previously described [41].

### 4.9. Statistical Analysis

All statistical analyses were performed using GraphPad Prism 5.0 (GraphPad Software, Inc., San Diego, CA, USA). Data analysis was performed using Student’s *t*-test, and statistical significance was defined as a *p*-value of less than 0.05.

## 5. Conclusions

In this study, following the observation that HER based mechanisms may be active in TNBC, our result shows varlitinib is able to suppress activation of ERK and Akt pathways and can exert significant anti-tumor activity in TNBC.

## Supplementary Materials

The following are available online at http://www.mdpi.com/2072-6694/11/1/105/s1, Figure S1: Varlitinib suppressed cell viability, Figure S2: Clinical significance of HER family in TNBC.
