# Supplementary material for: Varlitinib Downregulates HER/ERK Signaling and Induces Apoptosis in Triple Negative Breast Cancer Cells
Source: Cancers (Basel). 2019 Jan 17;11(1):105. doi: 10.3390/cancers11010105 (PMC6356324; doi:10.3390/cancers11010105)
Supplement: Supplementary file 1 [file cancers-11-00105-s001.pdf]

Supplementary Materials

Varlitinib Downregulates HER/ERK Signaling and Induces Apoptosis in Triple Negative Breast Cancer Cells

Chun-Yu Liu, Pei-Yi Chu, Chun-Teng Huang, Ji-Lin Chen, Hsiu-Ping Yang, Wan-Lun Wang, Ka-Yi Lau, Chia-Han Lee, Tien-Yun Lan, Tzu-Ting Huang, Po-Han Lin, Ming-Shen Dai and Ling-Ming Tseng

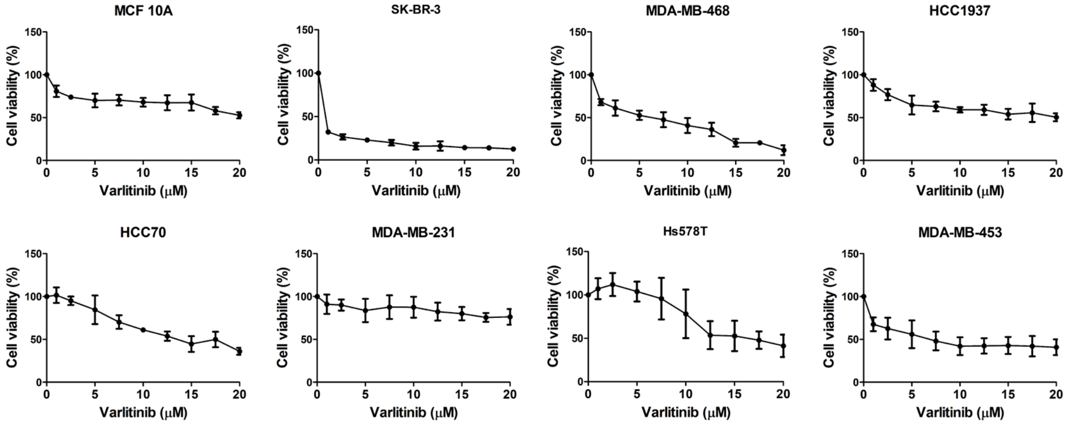

Figure S1. Varlitinib suppressed cell viability. MCF 10A, SK-BR-3, MDA-MB-468, HCC1937, HCC70, MDA-MB-231, Hs578T and MDA-MB-453 cells were treated with various concentrations of varlitinib for 72 h and cell viability was measured by MTT assay.

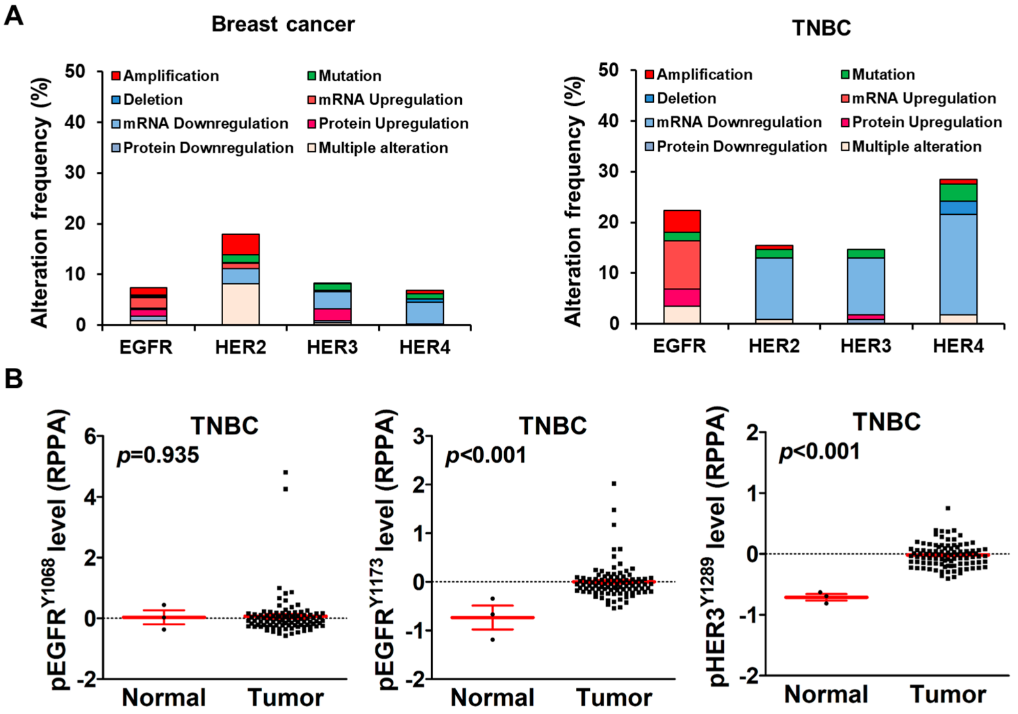

Figure S2. Clinical significance of HER family in TNBC. (**A**) EGFR, HER2, HER3 and HER4 gene alteration data from The Cancer Genome Atlas (TCGA) database, including copy number variation, mutation and mRNA dysregulation, in patients with breast cancer (left, N = 1098) and TNBC (right, N = 116) was analyzed. (**B**) Protein phosphorylation levels in TNBC and normal tissues were selected and examined from reverse phase protein arrays (RPPA) data in TCGA database.
